# Cost‐effectiveness analysis of a community‐based model for delivery of antiretroviral therapy to people with clinically stable HIV in Cambodia

**DOI:** 10.1002/jia2.26476

**Published:** 2025-07-07

**Authors:** Lo Yan Esabelle Yam, Pheak Chhoun, Ziya Tian, Michiko Nagashima‐Hayashi, Marina Zahari, Sovannary Tuot, Sovannarith Samreth, Bora Ngauv, Vichea Ouk, Kiesha Prem, Siyan Yi

**Affiliations:** ^1^ Saw Swee Hock School of Public Health National University of Singapore and National University Health System 12 Science Drive 2, #10‐01 Singapore 119260 Singapore; ^2^ KHANA Center for Population Health Research KHANA Phnom Penh Cambodia; ^3^ Department of Community and Global Health Graduate School of Medicine University of Tokyo Tokyo Japan; ^4^ National Center for HIV/AIDS, Dermatology and STD, Ministry of Health Phnom Penh Cambodia; ^5^ London School of Hygiene and Tropical Medicine University of London London UK

**Keywords:** people living with HIV, differentiated service delivery, multi‐month dispensing, community anti‐retroviral delivery, community intervention, Cambodia

## Abstract

**Introduction:**

In Cambodia, of all people living with HIV, 89% knew their status, 89% were receiving antiretroviral therapy (ART) and 87% had their viral load suppressed in 2023. In 2017, the national HIV programme introduced the multi‐month dispensing (MMD) model to reduce visits to ART clinics, thereby reducing the burden on people living with HIV and health facilities. A quasi‐experimental study introduced the community ART delivery (CAD) model, where community action workers (CAWs) delivered pre‐packaged antiretrovirals to their peers in the community. This study examined the cost‐effectiveness of the CAD compared to the MMD model.

**Methods:**

This study was conducted between 2021 and 2023 and involved 2040 stable people living with HIV in the CAD arm and 2049 in the MMD arm. Baseline and endline surveys included self‐reported ART adherence, quality of life, and medical and non‐medical expenses. Intention‐to‐treat analyses (ITTs) were conducted based on participants’ original treatment assignment, with multiple imputations performed for participants lost to follow‐up at the endline. Incremental cost‐effectiveness ratios (ICERs) on ART adherence and quality of life were generated using health system and societal perspectives. Cost‐effectiveness thresholds (CETs) were one‐time gross domestic product (GDP) per capita and opportunity cost.

**Results:**

Both arms observed a decline in ART adherence and good physical health, with a decline in CAD less than in the MMD (*p*‐value < 0.001). Similarly, a reduced proportion of participants reported good mental health across both arms; however, the difference was statistically insignificant. The ICERs for good physical health at the health system and societal levels were below the one‐time GDP per capita (Incremental Net Benefit = 77.49−83.03) but exceeded the opportunity cost CET. The ICERs for ART adherence at the health system and societal levels were above both CETs.

**Conclusions:**

The results showed that the CAD model was cost‐effective in reducing the decline in the physical health of people living with HIV during the COVID‐19 pandemic in Cambodia when a less stringent threshold was used. Further investigations are required to ascertain the cost‐effectiveness of the CAD model by factoring in the productivity gains within the health system.

**Clinical Trial Number:**

NCT04766710

## 1 INTRODUCTION

In 2023, 89% of people living with HIV in Cambodia knew their HIV status, 89% were receiving antiretroviral therapy (ART) and 87% had achieved viral load suppression [1, 2]. People living with HIV continue to face structural barriers such as internalised and experienced stigma that contribute to their fear of disclosing their HIV status and poorer mental and physical health [3]. Non‐disclosure of HIV status can impact their social support [4] and, altogether, affect their adherence to ART and retention in care [5, 6, 7, 8]. Lifelong treatment for HIV is another challenge in terms of time and resources [9]. HIV treatment in Cambodia is provided free of charge to people living with HIV. Since 2019, they have also been included in the Identification of Poor Households (IDPoor) mechanism, a national programme to support impoverished households. People living with HIV who are registered in the IDPoor receive an Equity Card, which entitles them to monthly stipends and free healthcare services under the Health Equity Fund (HEF) at public health facilities [10, 11]. However, transportation subsidies are no longer available under the revised HEF package [12]. Although transport is provided by some non‐governmental organizations (NGOs), the support is limited and does not cover all people living with HIV.

On the supply side, the Cambodian government grapples with an overstretched health workforce, particularly in rural areas [13, 14]. To reduce the burden on health facilities and people living with HIV, the World Health Organization (WHO) recommends ART differentiated service delivery (DSD) models [15]. DSD includes approaches such as multi‐month dispensing (MMD), a model introduced by the National Center for HIV/AIDS, Dermatology and STD (NCHADS) in 2017. Under the MMD model, stable people living with HIV receive antiretrovirals (ARVs) for a 3−6 month supply from ART clinics [16]. Another DSD model, the community ART delivery (CAD), was implemented and evaluated in Cambodia by the Khmer HIV/AIDS NGO Alliance (KHANA) from 2021 to 2023 [17]. The CAD model involved people living with HIV who served as community action workers (CAWs) in collecting pre‐packaged ARVs from ART clinics and distributing them to people living with HIV in their community action groups (CAGs) at monthly group sessions.

Additionally, CAWs provided medical support by monitoring vital signs and ART adherence and conducting health education and counselling with their members. Apart from medical support and ART delivery, the core of the CAD model is psychosocial support among people living with HIV through peer‐to‐peer interactions during the group sessions. The effectiveness of MMD and CAD models on people living with HIV and healthcare providers in the ART clinics was evaluated through the quasi‐experimental study [17]. This paper focuses on the cost‐effectiveness analysis of the CAD model by comparing it with the MMD model.

## 2 METHODS

### 2.1 Study design

The quasi‐experimental study involved 2040 people living with HIV in the CAD arm (intervention) and 2049 in the MMD arm (control). Before enrolment, people living with HIV were confirmed by clinicians to be clinically stable, based on the following eligibility criteria: (1) aged 15 years or older; (2) had been receiving first‐line ART for at least 1 year; (3) did not report ART‐related adverse reactions or drug interactions requiring regular monitoring; (4) were free from tuberculosis (either presumptive or confirmed) and other opportunistic infections; (5) were not taking any prophylactic treatment; (6) had good understanding of lifelong treatment and medication adherence; and (7) achieved at least two consecutive undetectable viral loads or CD4 counts above 200 cells/mm^3^. Pregnant or breastfeeding women were excluded from the study.

In the MMD arm, participants were recruited from 10 ART clinics in Phnom Penh, Kampong Cham, Pailin, Preah Sihanouk, Siem Reap and Prey Veng. Participants received 3−6 months of ARVs at each visit. In the CAD arm, participants were recruited from 10 other ART clinics in Phnom Penh, Kampong Thom, Kampot, Koh Kong and Takeo. Eighty‐two CAWs collected ARVs from the ART clinics and distributed them to their CAG members at monthly community meetings. All MMD and CAD arm participants visited the ART clinics for ad‐hoc consultation and required routine clinical review every 6 months. At the start of the study, CAWs received training from ART clinicians, pharmacists, counsellors and NCHADS programme staff on ART delivery, referral system and medication adherence to enable them to lead the community sessions. This study adhered to the Consolidated Health Economic Evaluation Reporting Standards (CHEERS) [18] (Supporting information).

### 2.2 Variables and measurements

Questionnaire surveys were planned for baseline (0 months), midline (12th month) and endline (24th month). However, the surveys were conducted only at two time points: baseline in October 2021 (0 months) and endline in May 2023 (19th month). The midline survey was not possible due to operational and budget challenges during the COVID‐19 pandemic. Trained data collectors administered the surveys, which captured participants’ socio‐demographic characteristics and self‐reported HIV treatment history and comorbidities. Outcomes of interest for the cost‐effectiveness analysis were self‐reported ART adherence and quality of life.

Self‐reported adherence has been recommended as a valid and reliable tool for research [19, 20]. The survey included five primary questions on ART adherence adapted from the Morisky Medication Adherence Scale [21, 22], widely used in HIV populations [23, 24, 25]. The questionnaire asked whether participants had missed any ARVs in the past 2 months, whether they found it difficult to remember taking ARVs, whether they sometimes stopped taking ARVs when they felt better, whether they had missed any doses in the past 4 days and whether they stopped taking ARVs when they felt worse. Two secondary items asked participants about the last time they missed taking ARVs and why they sometimes found it difficult to adhere to their ARV regimen. Participants were considered adherent if they answered “no” to all five primary questions.

Quality of life was measured using the Short‐Form‐12 (SF‐12) survey validated for use in people living with HIV [26, 27]. SF‐12 comprises 12 items representing eight health domains—physical function, social function, role limitations due to physical health, role limitations due to emotional problems, mental health, vitality, bodily pain and general health [28]. Responses to SF‐12 generated the Physical Component Score (PCS) and Mental Component Score (MCS). PCS higher than 50 indicates good physical health, and MCS higher than 42 indicates good mental health.

On costing, participants were asked to report on the medical (e.g. consultation) and non‐medical costs (e.g. transport) incurred by them and their caregivers when they travelled to the ART clinics. Separate questionnaire surveys were administered to the CAWs at the endline and the healthcare providers at the baseline and endline. In addition to questions on socio‐demographics, the survey for the CAWs included non‐medical costs incurred in their travel to the ART clinics and meetings with their CAG members. For the healthcare providers, the survey included questions on their roles, tasks, work characteristics and the time spent at the ART clinics.

### 2.3 Data analyses

A health economic analysis plan was developed to guide the evaluation. Analyses were performed in R (version 4.4.3) using the intention‐to‐treat (ITT) principle based on participants’ original treatment assignment. The time horizon was the intervention period, as outcomes were expected to occur during the study. Continuous variables were analysed for descriptive statistics using the two‐sample Student's *t*‐test or Mann‐Whitney U test when the normality assumption was not upheld. Pearson's Chi‐square or Fisher's exact test was used for categorical variables if the cell count was smaller than five.

To account for the loss‐to‐follow‐up (LTFU), multiple imputations were first performed using the fully conditional specification implemented by the MICE (multivariate imputation by chained equations) package under the assumption of *missing at random* [29, 30]. This approach was informed by observed associations between outcome variables, socio‐demographic characteristics and LTFU (Supporting information Table S1). Socio‐demographic characteristics and imputed variables, including outcomes and costs, were used as predictor variables to impute missing outcomes and costs of LTFU participants at the endline. The number of facility visits during the intervention period was imputed separately using socio‐demographic characteristics as predictors. The socio‐demographic characteristics of LTFU participants were assumed to remain unchanged from the baseline based on the rationale that they were likely stable over the 18‐month study period.

Within MICE, predictive mean matching was used for continuous variables, logistic regression for binary variables, proportional odds regression for ordinal variables and multinomial logistic regression for multi‐level categorical variables. Ten imputed datasets were generated using chained equations with 50 iterations for each multiple imputation. Convergence diagnostics was done to assess imputation stability.

Multivariable logistic regression was then used to assess the intervention effect on ART adherence and quality of life, adjusting for significantly different baseline covariates (*p* < 0.10) and those known to influence these outcomes [31, 32, 33, 34, 35, 36, 37, 38, 39, 40]. Nested models were compared to the full model using likelihood ratio tests, and model fit was assessed using Akaike Information Criterion and Bayesian Information Criterion. The final model selection prioritised parsimony and explanatory value. Final models were fitted in each imputed dataset and pooled using Rubin's rules [30]. Imputation uncertainty was assessed using the fraction of missing information (FMI) and variance components. FMI and the proportion of total variance due to between‐imputation variation were considered acceptable if they were below 0.25 [41].

Based on the final models, the average treatment effect for ART adherence and quality of life was estimated across imputed datasets using a difference‐in‐differences (DiD) approach [42, 43]. The final regression model was applied to each imputed dataset to predict the average outcomes of ART adherence and quality of life at baseline and endline for each study arm. The predicted probabilities were then pooled across imputations using Rubin's rules to account for within‐ and between‐imputation variance, incorporating uncertainty introduced by missing data. The DiD estimate was calculated as the difference in outcome change between endline and baseline between the intervention and control arms.

Health system and societal costs between October 2021 and April 2023 were estimated using a micro‐costing approach. Health system costs included salaries, training expenses and operational costs. Societal costs encompassed out‐of‐pocket expenditures (OOPEs) and productivity losses for people living with HIV, their caregivers and CAWs (Supporting information Table S2). All costs were adjusted for inflation using the Cambodia Consumer Price Index (CPI) to April 2024 [44] and converted to the United States Dollars (USD) at an exchange rate of 1 Riel = 0.00024 USD.

OOPE and productivity costs for people living with HIV were estimated using inverse probability weighting (IPW) to adjust for imbalances in baseline socio‐demographic characteristics between the intervention and control arms [45]. IPW was implemented by fitting a propensity score model using logistic regression, with treatment assignment as the outcome and socio‐demographic covariates as predictors. Predicted probabilities were bounded between 0.01 and 0.95, and stabilised IPW weights were then calculated and truncated at the 99th percentile to reduce the influence of extreme values. These weights were applied to the observed costs to derive weighted cost estimates. Weighted mean and total OOPE and productivity costs were computed within each treatment group and pooled across the imputed datasets using Rubin's rules to account for imputation‐related variance.

All cost items at both the health system and societal levels were aggregated to estimate the mean cost per participant for each study arm at each level. Incremental cost‐effectiveness ratios (ICERs) were then calculated by dividing the difference in mean costs per participant by the difference in the DiD of predicted outcome probabilities between the CAD and MMD models. ICERs were reported as cost per participant with good physical health and per ART‐adherent participant. The primary perspective was the health system, reflecting policymakers’ priorities, while the secondary perspective incorporated societal costs to capture broader economic implications. Two cost‐effectiveness thresholds (CETs) were applied: (1) one‐time Cambodia's gross domestic product (GDP) per capita in 2022 (USD 1799) and (2) a more stringent threshold of USD 304, based on opportunity cost estimates by Ochalek et al., both adjusted to April 2024 using Cambodia's CPI [46, 47].

### 2.4 Ethics

The National Ethics Committee for Health Research, Ministry of Health, Cambodia, approved this study (Reference: 258/NECHR). All participants provided written informed consent before data collection started.

## 3 RESULTS

**Figure 1:**
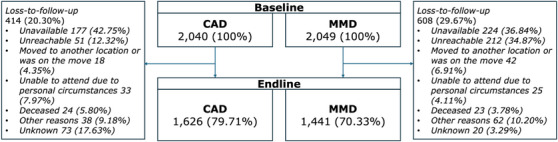
Number of participants in the MMD (control) and CAD (intervention) arms and reasons for loss‐to‐follow‐up. Abbreviations: CAD, community antiretroviral delivery; MMD, multi‐month dispensing. Number (%) of total sample size or *total loss‐to‐follow‐up*.

Among the 4089 participants, 1626 out of 2040 from the CAD arm (79.71%) and 1441 out of 2049 from the MMD arm (70.33%) remained at the endline (Figure 1).

### 3.1 Socio‐demographic characteristics

A higher proportion of participants in the MMD arm (77.11%) than in the CAD arm (61.42%) received HIV care in urban areas. Additionally, participants in the MMD arm were over four times more likely than those in the CAD arm (5.42% vs. 1.27%) to identify themselves as lesbian, gay, bisexual, transgender, queer, intersex, asexual or to prefer not to disclose their sexual orientation. Most participants were married, had a primary school education and were farmers or fishermen, self‐employed, or unemployed. Nearly twice as many participants in the MMD arm (19.42%) as in the CAD arm (10.74%) were diagnosed with HIV within the past 5 years. However, in both arms, the majority had been living with HIV and receiving ART for 11−20 years. The primary mode of transportation to ART clinics was motorised vehicles, including Tuk Tuks (trishaws), motorcycles or cars. Almost half of the participants in both arms reported spending less than 30 minutes travelling to ART clinics. Most remaining participants reported travel times of less than 2 hours. The median household income significantly differed between MMD and CAD arm participants. Furthermore, a significantly higher proportion of participants in the CAD arm (24.90%) reported having at least one comorbidity than those in the MMD arm (17.18%) (Table 1).

**Table 1:** Socio‐demographic characteristics of participants in the MMD (control) and CAD (intervention) arms

	MMD (*n* = 2049)	CAD (*n* = 2040)	*p‐*value
**ART clinic setting**			
Urban	1580 (77.11)	1253 (61.42)	< 0.001
Rural	469 (22.89)	787 (38.58)	
**Age groups**			
Young adults (15−24)	107 (5.22)	134 (6.57)	0.003
Adults (25−49)	1164 (56.81)	1046 (51.27)	
Older adults (50−64)	708 (34.55)	791 (38.77)	
Elderly (65+)	70 (3.42)	69 (3.38)	
**Gender groups**			
Male at birth	794 (38.75)	781 (38.28)	< 0.001
Female at birth	1144 (55.83)	1233 (60.44)	
LGBTQIA+	111 (5.42)	26 (1.27)	
**Marital status**			
Single	863 (42.12)	811 (39.75)	0.13
Married	1186 (57.88)	1229 (60.25)	
**Formal education**			
No formal education (0 years)	343 (16.74)	380 (18.63)	< 0.001
Primary school (1–6 years)	934 (45.58)	1040 (50.98)	
Secondary school (7–9 years)	443 (21.62)	404 (19.80)	
Tertiary (10–12 years)	235 (11.47)	151 (7.40)	
University (> 12 years)	94 (4.59)	65 (3.19)	
**Employment**			
Unemployed	356 (17.37)	411 (20.15)	< 0.001
Farmer/fisherman	501 (24.45)	571 (27.99)	
Motor/taxi driver	59 (2.88)	65 (3.19)	
Self‐employed business	428 (20.89)	338 (16.57)	
Government staff/uniformed officer	102 (4.98)	69 (3.38)	
Private employee/NGO staff	180 (8.78)	148 (7.25)	
Construction/factory worker	291 (14.20)	325 (15.93)	
Others	132 (6.44)	113 (5.54)	
**Family size**			
Living alone	45 (2.20)	54 (2.65)	0.01
Small family (2−4 members)	1421 (69.35)	1495 (73.28)	
Medium family (5−7 members)	515 (25.13)	442 (21.67)	
Large family (> 8 members)	68 (3.32)	49 (2.40)	
**Family with or without children**			
No children under 15	869 (42.41)	848 (41.57)	0.23
One child under 15	641 (31.28)	680 (33.33)	
Two children under 15	383 (18.69)	342 (16.76)	
> Three children under 15	156 (7.61)	170 (8.33)	
**Duration living with HIV**			
Newly diagnosed (1−5 years)	398 (19.42)	219 (10.74)	< 0.001
Medium duration (6−10 years)	392 (19.13)	325 (15.93)	
Long duration (11−15 years)	527 (25.72)	655 (32.11)	
Very long duration (16−20 years)	551 (26.89)	645 (31.62)	
Extensive duration (> 21 years)	181 (8.83)	196 (9.61)	
**Duration receiving ART therapy**			
Newly on ART (0−5 years)	445 (21.72)	277 (13.58)	< 0.001
Medium duration (6−10 years)	445 (21.72)	407 (19.95)	
Long duration (11−15 years)	625 (30.50)	724 (35.49)	
Very long duration (16−20 years)	469 (22.89)	556 (27.25)	
Extensive duration (> 21 years)	65 (3.17)	76 (3.73)	
**Mode of transportation to ART clinic**			
On foot/bicycle	71 (3.47)	82 (4.02)	< 0.001
Tuk tuk/motorcycle/car	1900 (92.73)	1946 (95.39)	
Boat/ship/others	78 (3.81)	12 (0.59)	
**Travel time to ART clinic**			
Short travel (< 0.5 hours)	855 (41.73)	944 (46.27)	0.005
Medium travel (> 0.5−2 hours)	985 (48.07)	918 (45.00)	
Long travel (> 2−8 hours)	198 (9.66)	160 (7.84)	
Very long travel (> 8 hours)	11 (0.54)	18 (0.88)	
**Other variables**			
Waiting time at ART clinic (Median [IQR], hour)	1.5 (1)	2 (2)	< 0.001
Household monthly income (Median [IQR], USD)	216 (240)	192 (237.6)	< 0.001
Has at least one co‐morbidity^a^	352 (17.18)	508 (24.90)	< 0.001

*Note*: Values in the table are numbers (%) unless indicated.

Abbreviations: ART, antiretroviral therapy; CAD, community antiretroviral delivery; IQR, interquartile range; LGBTQIA+, lesbian, gay, bisexual, transgender, queer, intersex, asexual or preferred not to reveal their orientation; MMD, multi‐month dispensing; NGO, non‐governmental organization; SD, standard deviation; USD, United States Dollar.

^a^ Co‐morbidity includes diabetes mellitus, high cholesterol and hypertension.

### 3.2 Intervention effects

ART adherence declined in both arms at endline (Table 2), with a less pronounced decline in the CAD arm compared to the MMD arm (AOR: 1.59, *p‐*value < 0.001) (Table 3). The estimated probability of ART adherence was 5 percentage points higher in the CAD arm than in the MMD arm (Table 4). Similarly, the proportion of participants reporting good physical health decreased in both arms (Table 2), with a greater decline in the MMD arm (AOR:1.64, *p‐*value < 0.001) (Table 3). The estimated probability of good physical health was 11 percentage points higher in the CAD arm than in the MMD arm (Table 4). A lower proportion of participants in both arms reported good mental health at endline (Table 2), with no significant difference between arms (AOR: 1.21, *p‐*value = 0.10) (Table 3).

**Table 2:** ART adherence and quality of life of participants at baseline and endline in the MMD (control) and CAD (intervention) arms

	Baseline			Endline		
	MMD (*n* = 2049) *n* (%)	CAD (*n* = 2040) *n* (%)	*p‐*value	MMD (*n* = 2049) *n* (%)	CAD (*n* = 2040) *n* (%)	*p‐*value
**ART adherence**						
ART adherent	1840 (89.80)	1766 (86.60)	0.002	1635 (79.78)	1680 (82.34)	0.04
Non‐ART adherent	209 (10.20)	274 (13.43)		414 (20.22)	360 (17.66)	
**Quality of life – physical health**						
Good physical health (PCS ≥ 50)	1431 (69.84)	1128 (55.29)	< 0.001	1020 (49.78)	947 (46.41)	0.03
Poor physical health (PCS < 50)	618 (30.16)	912 (44.71)		1029 (50.23)	1093 (53.59)	
**Quality of life – mental health**						
Good mental health (MCS ≥ 42)	1706 (83.26)	1557 (76.32)	< 0.001	1605 (78.33)	1520 (74.53)	0.004
Poor mental health (MCS < 42)	343 (16.74)	483 (23.68)		444 (21.67)	520 (25.47)	

Abbreviations: ART, antiretroviral therapy; CAD, community antiretroviral delivery; MCS, Mental Component Score; MMD, multi‐month dispensing; PCS, Physical Component Score.

**Table 3:** Intervention effect on ART adherence and quality of life

	Crude		Adjusted model	
	OR (95% CI)	*p‐*value	AOR (95% CI)	*p‐*value
ART adherent	1.61 (1.24, 2.10)	< 0.001	1.59 (1.22, 2.08)^a^	< 0.001
Good physical health	1.64 (1.36, 1.97)	< 0.001	1.64 (1.35, 1.99)^b^	< 0.001
Good mental health	1.25 (1.00, 1.56)	0.05	1.21 (0.97, 1.52)^c^	0.10

Abbreviations: AOR, adjusted odds ratio; ART, antiretroviral therapy; CI, confidence interval; OR, odds ratio.

^a^ Covariates included for adjustment: ART site setting, age, employment and duration of receiving anti‐retroviral treatment.

^b^ Covariates included for adjustment: Gender, age, formal education, employment, travel time, monthly household income and presence of comorbidity.

^c^ Covariates included for adjustment: ART site setting, gender, formal education, employment, size of family, travel time, monthly household income, duration receiving anti‐retroviral treatment and presence of comorbidity.

**Table 4:** Difference‐in‐difference of predicted probabilities of participants adhering to ART and having good physical health between the MMD (control) and CAD (intervention) arms, accounting for baseline differences

	MMD (*n* = 2049)			CAD (*n* = 2040)			
	Baseline Mean (95% CI)	Endline Mean (95% CI)	Diff (A)	Baseline Mean (95% CI)	Endline Mean (95% CI)	Diff (B)	DiD (B−A)
ART adherent	0.91 (0.89, 0.92)	0.81 (0.78, 0.84)	−0.10	0.87 (0.84, 0.89)	0.82 (0.79, 0.85)	−0.05	0.05
Good physical health	0.65 (0.62, 0.68)	0.43 (0.40, 0.47)	−0.22	0.51 (0.47, 0.54)	0.42 (0.38, 0.45)	−0.09	0.11

Abbreviations: ART, antiretroviral therapy; CAD, community antiretroviral delivery; CI, confidence interval; DiD, difference in difference; MMD, multi‐month dispensing.

### 3.3 Cost‐effectiveness

Most participants did not incur any medical expenses at baseline and endline. However, expenses as high as 400,000 Riel (USD 96) were reported for other medical costs, including service fees, charges for extended ARV supplies, additional medications, laboratory tests and informal fees requested by doctors for “noodles and coffee” (Table 5). Transport and food were the primary contributors of OOPE in both arms (Table 5). On average, participants in the MMD arm made 5.25 trips to the ART clinics over the study period, slightly higher than the 4.72 times in the CAD arm (*p‐*value < 0.001). The trip reduction among CAD participants translated into time and cost savings, contributing to lower OOPE and productivity gains (Table 6). The ICERs for achieving good physical health per participant were $1094.55 at the health system and $1044.18 at the societal levels. These ICERs fell below the CET of one‐time Cambodia's GDP per capita (incremental net benefit = 77.49−83.03) but exceeded the opportunity cost CET. In contrast, all other ICERs, including the ICER per ART‐adherent participant at the health system ($2408) and societal levels ($2297.2), exceeded both CETs (Table 7).

**Table 5:** Medical and non‐medical costs incurred by people living with HIV and their caregivers on their last trip to the ART clinic in the MMD (control) and CAD (intervention) arms (in Riel, unadjusted)

	Baseline			Endline		
	MMD (*n* = 2049) Median (range)	CAD (*n* = 2040) Median (range)	*p‐*value	MMD (*n* = 1441) Median (range)	CAD (*n* = 1626) Median (range)	*p‐*value
**Medical cost**						
Consultation fee	0 (60,000)	0 (0)	0.05	0 (100,000)	0 (100,000)	0.29
Diagnostic fee	0 (40,000)	0 (40,000)	0.42	0 (10,000)	0 (20,000)	0.33
Medicine fee	0 (60,000)	0 (5000)	< 0.001	0 (300,000)	0 (5000)	0.11
Other medical cost	0 (40,000)	0 (400,000)	0.05	0 (15,000)	0 (10,000)	0.98
Total direct cost	0 (80,000)	0 (400,000)	< 0.001	0 (400,000)^a^	0 (100,000)^b^	0.47
**Non‐medical cost**						
Transport cost	8000 (1,400,000)	6000 (230,000)	< 0.001	10,000 (400,000)	10,000 (2,000,000)	< 0.001
Food cost	5000 (80,000)	5000 (80,000)	0.47	5000 (230,000)	5000 (200,000)	0.28
Lodging cost	0 (60,000)	0 (90,000)	0.99	0 (40,000)	0 (28,000)	0.17
Other non‐medical cost	0 (40,000)	0 (80,000)	< 0.001	0 (60,000)	0 (50,000)	0.77
Total indirect cost	14,500 (1,410,000)	12,450 (247,000)	< 0.001	15,000 (605,000)^c^	15,000 (2,200,000)^d^	< 0.001
Total OOPE	15,000 (1,410,000)	12,900 (402,000)	< 0.001	15,000 (800,000)^e^	15,000 (2,200,000)^f^	< 0.001

Abbreviations: CAD, community antiretroviral delivery; LTFU, loss to follow‐up; MMD, multi‐month dispensing; OOPE, out‐of‐pocket expenditure.

^a^ The median (range) total direct cost for all participants in MMD, including imputed costs of those LTFU at the endline, is 0 (400,000).

^b^ The median (range) total direct cost for all participants in CAD, including imputed costs of those LTFU at the endline, is 0 (100,000).

^c^ The median (range) total indirect cost for all participants in MMD, including imputed costs of those LTFU at the endline, is 15,000 (1,090,600).

^d^ The median (range) total indirect cost for all participants in CAD, including imputed costs of those LTFU at the endline, is 15,000 (2,200,000).

^e^ The median (range) total OOPE for all participants in MMD, including imputed costs of those LTFU at the endline, is 15,200 (1,166,000).

^f^ The median (range) total OOPE for all participants in CAD, including imputed costs of those LTFU at the endline, is 15,000 (2,200,000).

**Table 6:** Total cost incurred at the health system and societal levels in the MMD (control) and CAD (intervention) arms (in USD and adjusted for inflation)

	MMD (*n* = 2049)	CAD (*n* = 2040)
**Health system cost**		
CAW salary	−	235,260
Training	4733.30	6007.37
Meetings with CAWs and communication cost for ART clinics to communicate with CAWs		9055.03
**Total health system cost (primary perspective)**	4733.30	250,322.40
**Mean cost/person living with HIV**	2.31	122.71
**Societal cost**		
OOPE (people living with HIV and their caregivers)^a^	58,764.57	51,008.84
OOPE (CAW)	0	135,891.18
Productivity cost (people living with HIV)	21,910.09	18,007.79
Productivity cost (CAW)	0	85,991.17
Subtotal cost	80,674.66	290,898.98
**Total health system and societal cost (secondary perspective)**	85,407.96	319,339.03^b^
**Mean cost/person living with HIV (secondary perspective)**	41.68	156.54

Abbreviations: CAD, community antiretroviral delivery; CAW, community action worker; MMD, multi‐month dispensing; OOPE, out‐of‐pocket expenditure.

^a^ At the baseline, a total of 144 (93 in the CAD [intervention] and 51 in the MMD [control]), and at the endline, a total of 176 (91 in the CAD [intervention] and 85 in the MMD [control]) brought a caregiver for their last visit to the ART facility. Expenses incurred by their caregiver are included in the OOPE cost reported in this table.

^b^ Total health system and societal costs exclude OOPE and productivity losses incurred by CAWs, as their salary, transport and communication costs were covered by the health system.

**Table 7:** Incremental cost‐effectiveness ratios of the difference in cost and outcomes in ART adherence and quality of life between the MMD (control) and CAD (intervention) arms

				CET (1): One‐time GDP per capita (USD1799)	CET (2): Opportunity cost (USD304)
	Incremental cost	Incremental effect	ICER	INB	INB
**ART adherence**					
Health system	120.40	0.05	2408	−30.45	−105.2
Societal	114.86	0.05	2297.2	−24.91	−99.66
**Good physical health**					
Health system	120.40	0.11	1094.55	77.49	−86.96
Societal	114.86	0.11	1044.18	83.03	−81.42

Abbreviations: ART, antiretroviral therapy; CET, cost‐effectiveness threshold; ICER, incremental cost‐effectiveness ratio; INB, incremental net benefit.

## 4 DISCUSSION

The analyses demonstrated the cost‐effectiveness of the CAD model in mitigating the decline in good physical health at both health system and societal levels when evaluated against the CET of one‐time Cambodia's GDP per capita. However, the CAD model was not cost‐effective when assessed using the more stringent opportunity cost CET. Notably, there was a decline in good physical and mental health and ART adherence across both arms. As the study coincided with the third phase of the COVID‐19 pandemic in Cambodia, the decline in ART adherence and quality of life could be due to the direct and indirect impacts of the pandemic [48]. During the pandemic, essential health services and ARV supplies were disrupted, and the health workforce, already overstretched before the pandemic, struggled with an even heavier workload due to additional COVID‐19‐related responsibilities [49, 50, 51, 52, 53, 54]. These disruptions impacted access to ART, and fear of visiting health facilities, financial hardships and caregiving duties also affected the use of HIV services [52]. Although not definitive due to conflicting study results, reviews have indicated that people living with HIV have a higher likelihood of experiencing severe COVID‐19 [55], increased mortality [55, 56] and higher rates of hospitalisation from COVID‐19 [57]. These factors could affect ART adherence and the quality of life of people living with HIV, potentially explaining the deteriorated outcomes observed in this study.

Social distancing and COVID‐19 restrictions necessitated several adaptations in this study. For instance, community workers in the MMD sites have been reported to deliver ARVs to people living with HIV to support continued treatment. In the CAD arm, on some occasions, CAGs had to meet in smaller groups, or CAWs had to deliver ARVs to their members’ houses rather than meet as a group. These adaptations might have affected the peer‐to‐peer support envisaged for the CAD model. Though necessary during the pandemic, these deviations from the protocol potentially account for the small difference in the number of visits to the ART clinics made by participants in the MMD and CAD arms. This suggests that the cost‐effectiveness of the CAD model might have been underestimated, as the differences in the OOPE and productivity losses for participants between the two models might have been more significant if not for these protocol deviations.

Altogether, despite the protocol deviations, the CAD model has shown mitigating effects on the drop in ART adherence and quality of life of people living with HIV during the COVID‐19 pandemic. These findings support earlier studies that showed that the CAD model had delivered comparable or better outcomes than facility‐based services in ART adherence, viral load suppression, retention in care and HIV‐related mortality [58, 59]. Although the effectiveness of DSD, such as MMD and CAD models, has been studied in Asia and Africa [60, 61, 62, 63, 64], there is a lack of cost‐effectiveness studies on DSD models. Most literature focuses on cost analysis [65, 66, 67]. This cost‐effectiveness study contributes to the literature on the economic evaluation of DSD and, more broadly, on the economic evaluation of HIV, which has seen an increase in publications but is concentrated in developed countries [68].

Another interesting finding is the consultation fee, informal service fees, charges for more extended ARV supplies and other payments requested by doctors reported in this study, reinforcing previous reports of unofficial charges for HIV services [69]. In addition, transport costs, similar to other studies in Indonesia and Vietnam, made up the bulk of OOPE for HIV treatment [70, 71]. Considering that cost is a barrier to care, especially for people living with HIV who often face socio‐economic hardships and are more likely to be from the lower socio‐economic strata [72], future studies should explore the effect of these OOPEs on retention in the HIV care continuum in low‐ and middle‐income country settings.

This study has several limitations. First, the absence of midline data limited the verification of outcome trends, which may not accurately reflect the true intervention effect and could instead indicate regression towards the mean. Additionally, the assumption of parallel trends required for the DiD method could not be validated due to the use of data from only two time points. Second, the time participants spent with the CAWs in the CAD model was not quantified. Therefore, the productivity gains from reduced time at the ART clinics might be overestimated, inflating the CAD model's cost‐effectiveness. Third, adherence to routine viral load tests was challenging during the pandemic. Consequently, self‐reported adherence was used, which is subject to recall bias and social desirability bias, potentially resulting in over‐reporting. Furthermore, the Morisky Medication Adherence Scale, though widely used in HIV populations, has not been validated specifically for people living with HIV, and its reliability as a measure of adherence for HIV populations remains uncertain.

Fourth, the LTFU participants were imputed under their assigned study arms consistent with the ITT principle. However, some LTFU participants may have died during the study period. As a result, imputing outcomes for deceased participants could either underestimate or overestimate the estimated intervention effect, depending on the distribution of unobserved mortality among LTFU across study arms. Lastly, while improvements in healthcare provider productivity were of interest, the data lacked the granularity to quantify productivity gains in the health system from the CAD model. Field visit feedback revealed that healthcare workers at CAD sites redirected the time saved to people living with HIV who required more attention. Further investigation is needed to validate the cost‐effectiveness of the CAD model, considering productivity gains and improvements in quality of care within the health system.

## 5 CONCLUSIONS

The CAD model helped mitigate the decline in ART adherence and quality of life of stable people living with HIV during the COVID‐19 pandemic and was cost‐effective in reducing the drop in physical health when evaluated against a less stringent threshold. However, further studies are needed to validate the cost‐effectiveness of the CAD model.

## COMPETING INTERESTS

The authors declare that they have no competing interests.

## AUTHORS’ CONTRIBUTIONS

LYEY contributed to the study design, developed the data analysis plan, conducted the data analyses and wrote the draft. PC contributed to the study design, led the data collection and provided input on the draft. ZT, MN‐H and MZ supported the data analyses and provided feedback on the draft. SS, BN and VO contributed to the study design and offered strategic advice on the study's implementation, ensuring alignment with the national strategic plan for HIV/AIDS and prioritising the needs of beneficiaries in the study's implementation. ST and KP contributed to the study design. KP supported the data analyses and the draft. SY led the overall study, encompassing study design, implementation, data collection, analyses and finalising the draft. All authors have read and approved the final manuscript.

## FUNDING

The study received the funding support from L' Initiative through Expertise France (Grant No. 19‐SB0765). The funder has no role in the study design, project implementation and evaluation, and manuscript writing.

## Supporting information




**Supporting information**: CHEERS 2022 Checklist.
**Table S1**: Univariate regression analysis of loss to follow‐up with outcomes and sociodemographic characteristics at baseline.
**Table S2**: Computation of cost items at the health system and societal levels.

## Data Availability

The data that support the findings of this study are available on request from the corresponding author. The data are not publicly available due to privacy or ethical restrictions.

## References

[1] Global AIDS Monitoring 2019: Country Progress Report—Cambodia. Geneva: UNAIDS; 2019.

[2] Cambodia: HIV Country Profile 2024. Geneva: World Health Organization; 2024.

[3] Yi S , Chhoun P , Suong S , Thin K , Brody C , Tuot S . AIDS‐related stigma and mental disorders among people living with HIV: a cross‐sectional study in Cambodia. PLoS One. 2015;10(3):e0121461. 25806534 10.1371/journal.pone.0121461 PMC4373790

[4] Smith R , Rossetto K , Peterson BL . A meta‐analysis of disclosure of one's HIV‐positive status, stigma and social support. AIDS Care. 2008;20(10):1266–1275. 18608080 10.1080/09540120801926977

[5] Sweeney SM , Vanable PA . The association of HIV‐related stigma to HIV medication adherence: a systematic review and synthesis of the literature. AIDS Behav. 2016;20(1):29–50. 26303196 10.1007/s10461-015-1164-1

[6] Katz IT , Ryu AE , Onuegbu AG , Psaros C , Weiser SD , Bangsberg DR , et al. Impact of HIV‐related stigma on treatment adherence: systematic review and meta‐synthesis. J Int AIDS Soc. 2013;16(3 Suppl 2):18640. 24242258 10.7448/IAS.16.3.18640 PMC3833107

[7] Kane JC , Elafros MA , Murray SM , Mitchell EMH , Augustinavicius JL , Causevic S , et al. A scoping review of health‐related stigma outcomes for high‐burden diseases in low‐ and middle‐income countries. BMC Med. 2019;17(1):17. 30764819 10.1186/s12916-019-1250-8 PMC6376728

[8] IN DANGER: UNAIDS Global AIDS Update 2022. Geneva: Joint United Nations Programme on HIV/AIDS; 2022.

[9] Buell KG , Chung C , Chaudhry Z , Puri A , Nawab K , Ravindran RP . Lifelong antiretroviral therapy or HIV cure: the benefits for the individual patient. AIDS Care. 2016;28(2):242–246. 26357912 10.1080/09540121.2015.1074653

[10] Breaking barriers: Cambodia's inclusive approach to HIV social protection. Phnom Penh: Khmer Times; 2023. Available from: https://www.khmertimeskh.com/501411892/breaking‐barriers‐cambodias‐inclusive‐approach‐to‐hiv‐social‐protection/

[11] Kongnov T . Govt aims for 100% Health Equity Card coverage for HIV sufferers. Phnom Penh: Khmer Times; 2024. Available from: https://www.khmertimeskh.com/501485277/govt‐aims‐for‐100‐health‐equity‐card‐coverage‐for‐hiv‐sufferers/

[12] Jain BMS . Expanding Health Equity Fund coverage for people living with HIV in Cambodia: costing and policy options. Washington, DC; 2020.

[13] Kobashi Y , Chou K , Slaiman N , Neun P , Hayashi Y , Tsubokura M , et al. Improving the rural‐urban balance in Cambodia's health services. Int J Health Policy Manag. 2021;10(6):358–359. 32729285 10.34172/ijhpm.2020.136 PMC9056147

[14] Vun MC , Fujita M , Rathavy T , Eang MT , Sopheap S , Sovannarith S , et al. Achieving universal access and moving towards elimination of new HIV infections in Cambodia. J Int AIDS Soc. 2014;17(1):18905. 24950749 10.7448/IAS.17.1.18905 PMC4065309

[15] Consolidated guidelines on person‐centred HIV strategic information: strengthening routine data for impact. Policy brief on integrating and strengthening monitoring of differentiated ART service delivery. Geneva: World Health Organization; 2023.

[16] Standard operating procedure on appointment‐spacing and multi‐month dispensing (MMD) in Cambodia. Phnom Penh: Ministry of Health; 2023.

[17] Tuot S , Teo AKJ , Prem K , Chhoun P , Pall C , Ung M , et al. Community‐based model for the delivery of antiretroviral therapy in Cambodia: a quasi‐experimental study protocol. BMC Infect Dis. 2021;21(1):763. 34362310 10.1186/s12879-021-06414-y PMC8344198

[18] Husereau D , Drummond M , Augustovski F , de Bekker‐Grob E , Briggs AH , Carswell C , et al. Consolidated Health Economic Evaluation Reporting Standards 2022 (CHEERS 2022) Statement: updated reporting guidance for health economic evaluations. Value Health. 2022;25(1):3–9. 35031096 10.1016/j.jval.2021.11.1351

[19] Arnsten JH , Demas PA , Farzadegan H , Grant RW , Gourevitch MN , Chang CJ , et al. Antiretroviral therapy adherence and viral suppression in HIV‐infected drug users: comparison of self‐report and electronic monitoring. Clin Infect Dis. 2001;33(8):1417–1423. 11550118 10.1086/323201 PMC2692641

[20] Simoni JM , Kurth AE , Pearson CR , Pantalone DW , Merrill JO , Frick PA . Self‐report measures of antiretroviral therapy adherence: a review with recommendations for HIV research and clinical management. AIDS Behav. 2006;10(3):227–245. 16783535 10.1007/s10461-006-9078-6 PMC4083461

[21] Morisky DE , Green LW , Levine DM . Concurrent and predictive validity of a self‐reported measure of medication adherence. Med Care. 1986;24(1):67–74. 3945130 10.1097/00005650-198601000-00007

[22] Krousel‐Wood M , Islam T , Webber LS , Re RN , Morisky DE , Muntner P . New medication adherence scale versus pharmacy fill rates in seniors with hypertension. Am J Manag Care. 2009;15(1):59–66. 19146365 PMC2728593

[23] Ruanjahn G , Roberts D , Monterosso L . An exploration of factors influencing adherence to highly active anti‐retroviral therapy (HAART) among people living with HIV/AIDS in Northern Thailand. AIDS Care. 2010;22(12):1555–1561. 20582752 10.1080/09540121003759901

[24] Biney IJK , Kyei KA , Ganu VJ , Kenu E , Puplampu P , Manortey S , et al. Antiretroviral therapy adherence and viral suppression among HIV‐infected adolescents and young adults at a tertiary hospital in Ghana. Afr J AIDS Res. 2021;20(4):270–276. 34905452 10.2989/16085906.2021.1998783

[25] Adu C , Mensah KA , Ahinkorah BO , Osei D , Tetteh AW , Seidu AA . Socio‐demographic factors associated with medication adherence among people living with HIV in the Kumasi Metropolis, Ghana. AIDS Res Ther. 2022;19(1):50. 36376918 10.1186/s12981-022-00474-z PMC9662109

[26] Chariyalertsak S , Wansom T , Kawichai S , Ruangyuttikarna C , Kemerer VF , Wu AW . Reliability and validity of Thai versions of the MOS‐HIV and SF‐12 quality of life questionnaires in people living with HIV/AIDS. Health Qual Life Outcomes. 2011;9:15. 21406088 10.1186/1477-7525-9-15 PMC3068932

[27] Patel AR , Lester RT , Marra CA , van der Kop ML , Ritvo P , Engel L , et al. The validity of the SF‐12 and SF‐6D instruments in people living with HIV/AIDS in Kenya. Health Qual Life Outcomes. 2017;15(1):143. 28716065 10.1186/s12955-017-0708-7 PMC5513113

[28] Ware JE , Kosinski M , Keller SD . A 12‐item short‐form health survey: construction of scales and preliminary tests of reliability and validity. Med Care. 1996;34(3):220–233. 8628042 10.1097/00005650-199603000-00003

[29] Xi W , Pennell ML , Andridge RR , Paskett ED . Comparison of intent‐to‐treat analysis strategies for pre‐post studies with loss to follow‐up. Contemp Clin Trials Commun. 2018;11:20–29. 30023456 10.1016/j.conctc.2018.05.008 PMC6022256

[30] van Buuren S , Groothuis‐Oudshoorn K . mice: multivariate imputation by chained equations in R. J Stat Softw. 2011;45(3):1–67.

[31] Tuot S , Sim JW , Nagashima‐Hayashi M , Chhoun P , Teo AKJ , Prem K , et al. What are the determinants of antiretroviral therapy adherence among stable people living with HIV? A cross‐sectional study in Cambodia. AIDS Res Ther. 2023;20(1):47. 37452342 10.1186/s12981-023-00544-w PMC10347818

[32] de los Rios P , Okoli C , Punekar Y , Allan B , Muchenje M , Castellanos E , et al. Prevalence, determinants, and impact of suboptimal adherence to HIV medication in 25 countries. Prev Med. 2020;139:106182. 32593732 10.1016/j.ypmed.2020.106182

[33] Wasti SP , van Teijlingen E , Simkhada P , Randall J , Baxter S , Kirkpatrick P , et al. Factors influencing adherence to antiretroviral treatment in Asian developing countries: a systematic review. Trop Med Int Health. 2012;17(1):71–81. 21967241 10.1111/j.1365-3156.2011.02888.x

[34] Bijker R , Jiamsakul A , Kityo C , Kiertiburanakul S , Siwale M , Phanuphak P , et al. Adherence to antiretroviral therapy for HIV in sub‐Saharan Africa and Asia: a comparative analysis of two regional cohorts. J Int AIDS Soc. 2017;20(1):21218. 28362063 10.7448/IAS.20.1.21218 PMC5467608

[35] Xuan Tran B , Thanh Nguyen L , Hoang Nguyen N , Van Hoang Q . Determinants of antiretroviral treatment adherence among HIV/AIDS patients: a multisite study. Glob Health Action. 2013;6(1):19570. 23497956 10.3402/gha.v6i0.19570 PMC3600425

[36] Tran BX , Ohinmaa A , Nguyen LT , Nguyen TA , Nguyen TH . Determinants of health‐related quality of life in adults living with HIV in Vietnam. AIDS Care. 2011;23(10):1236–1245. 21711211 10.1080/09540121.2011.555749

[37] Ghiasvand H , Higgs P , Noroozi M , Ghaedamini Harouni G , Hemmat M , Ahounbar E , et al. Social and demographical determinants of quality of life in people who live with HIV/AIDS infection: evidence from a meta‐analysis. Biodemography Soc Biol. 2020;65(1):57–72. 30882251 10.1080/19485565.2019.1587287

[38] Khumsaen N , Aoup‐por W , Thammachak P . Factors influencing quality of life among people living with HIV (PLWH) in Suphanburi Province, Thailand. J Assoc Nurses AIDS Care. 2012;23(1):63–72. 21497112 10.1016/j.jana.2011.01.003

[39] Rayanakorn A , Ong‐artborirak P , Ademi Z , Chariyalertsak S . Predictors of stigma and health‐related quality of life among people living with HIV in Northern Thailand. AIDS Patient Care STDs. 2022;36(5):186–193. 35507323 10.1089/apc.2022.0035 PMC9125577

[40] Yang Y , Thai S , Choi J . An evaluation of quality of life among Cambodian adults living with HIV/AIDS and using antiretroviral therapy: a short report. AIDS Care. 2016;28(12):1546–1550. 27285879 10.1080/09540121.2016.1192100

[41] White IR , Royston P , Wood AM . Multiple imputation using chained equations: issues and guidance for practice. Stat Med. 2011;30(4):377–399. 21225900 10.1002/sim.4067

[42] Zhou H , Taber C , Arcona S , Li Y . Difference‐in‐differences method in comparative effectiveness research: utility with unbalanced groups. Appl Health Econ Health Policy. 2016;14(4):419–429. 27371369 10.1007/s40258-016-0249-y PMC4937082

[43] Muller CJ , MacLehose RF . Estimating predicted probabilities from logistic regression: different methods correspond to different target populations. Int J Epidemiol. 2014;43(3):962–970. 24603316 10.1093/ije/dyu029 PMC4052139

[44] Cambodia Consumer Price Index CPI. Phnom Penh: Trading Economics. (accessed 28/12/2024). Available from: https://tradingeconomics.com/cambodia/consumer‐price‐index‐cpi.

[45] Chesnaye NC , Stel VS , Tripepi G , Dekker FW , Fu EL , Zoccali C , et al. An introduction to inverse probability of treatment weighting in observational research. Clin Kidney J. 2022;15(1):14–20. 35035932 10.1093/ckj/sfab158 PMC8757413

[46] Ochalek J , Lomas J , Claxton K . Estimating health opportunity costs in low‐income and middle‐income countries: a novel approach and evidence from cross‐country data. BMJ Glob Health. 2018;3(6):e000964. 10.1136/bmjgh-2018-000964 PMC6231096 30483412

[47] Revill P , Ochalek J , Lomas J , Nakamura R , Woods B , Rollinger A , et al. Cost‐effectiveness thresholds: guiding health care spending for population health improvement. York: University of York Economics; 2015.

[48] Srean C , Grace K , Sovathiro M , Willem Van De P , Wim Van D , Por I , et al. Descriptive assessment of COVID‐19 responses and lessons learnt in Cambodia, January 2020 to June 2022. BMJ Glob Health. 2023;8(5):e011885. 10.1136/bmjgh-2023-011885 PMC10163327 37137538

[49] Downey LE , Gadsden T , Vilas VDR , Peiris D , Jan S . The impact of COVID‐19 on essential health service provision for endemic infectious diseases in the South‐East Asia region: a systematic review. Lancet Reg Health—Southeast Asia. 2022;1:100011. 35769109 10.1016/j.lansea.2022.04.007 PMC9069250

[50] Mirzaei H , Moradi Y , Abbaszadeh S , Nasiri N , Mehmandoost S , Khezri M , et al. The impact of COVID‐19 on disruptions of HIV‐related services: a rapid review. Med J Islam Repub Iran. 2022;36:98. 36419948 10.47176/mjiri.36.98 PMC9588155

[51] 2020 Global AIDS Update ⁠— Seizing the moment ⁠— Tackling entrenched inequalities to end epidemics. Geneva: UNAIDS; 2020.

[52] Seang K , Ky S , Ngauv B , Mam S , Ouk V , Saphonn V . Using relational community engagement within the Digital Health Intervention (DHI) to improve access and retention among people living with HIV (PLWH): findings from a mixed‐method study in Cambodia. Int J Environ Res Public Health. 2023;20(7):5247. 37047863 10.3390/ijerph20075247 PMC10093806

[53] Rick F , Odoke W , van den Hombergh J , Benzaken AS , Avelino‐Silva VI . Impact of coronavirus disease (COVID‐19) on HIV testing and care provision across four continents. HIV Med. 2022;23(2):169–177. 34632685 10.1111/hiv.13180 PMC8653012

[54] SeyedAlinaghi S , Mirzapour P , Pashaei Z , Afzalian A , Tantuoyir MM , Salmani R , et al. The impacts of COVID‐19 pandemic on service delivery and treatment outcomes in people living with HIV: a systematic review. AIDS Res Ther. 2023;20(1):4. 36609313 10.1186/s12981-022-00496-7 PMC9821373

[55] Oyelade T , Alqahtani JS , Hjazi AM , Li A , Kamila A , Raya RP . Global and regional prevalence and outcomes of COVID‐19 in people living with HIV: a systematic review and meta‐analysis. Trop Med Infect Dis. 2022;7(2):22. 35202217 10.3390/tropicalmed7020022 PMC8880028

[56] Ssentongo P , Heilbrunn ES , Ssentongo AE , Advani S , Chinchilli VM , Nunez JJ , et al. Epidemiology and outcomes of COVID‐19 in HIV‐infected individuals: a systematic review and meta‐analysis. Sci Rep. 2021;11(1):6283. 33737527 10.1038/s41598-021-85359-3 PMC7973415

[57] Puyat JH , Fowokan A , Wilton J , Janjua NZ , Wong J , Grennan T , et al. Risk of COVID‐19 hospitalization in people living with HIV and HIV‐negative individuals and the role of COVID‐19 vaccination: a retrospective cohort study. Int J Infect Dis. 2023;135:49–56. 37419410 10.1016/j.ijid.2023.06.026

[58] Nachega JB , Adetokunboh O , Uthman OA , Knowlton AW , Altice FL , Schechter M , et al. Community‐based interventions to improve and sustain antiretroviral therapy adherence, retention in HIV care and clinical outcomes in low‐ and middle‐income countries for achieving the UNAIDS 90‐90‐90 targets. Curr HIV/AIDS Rep. 2016;13(5):241–255. 27475643 10.1007/s11904-016-0325-9 PMC5357578

[59] Limbada M , Zijlstra G , Macleod D , Ayles H , Fidler S . A systematic review of the effectiveness of non‐health facility based care delivery of antiretroviral therapy for people living with HIV in sub‐Saharan Africa measured by viral suppression, mortality and retention on ART. BMC Public Health. 2021;21(1):1110. 34112135 10.1186/s12889-021-11053-8 PMC8194040

[60] Roy M , Bolton Moore C , Sikazwe I , Holmes CB . A review of differentiated service delivery for HIV treatment: effectiveness, mechanisms, targeting, and scale. Curr HIV/AIDS Rep. 2019;16(4):324–334. 31230342 10.1007/s11904-019-00454-5

[61] Lujintanon S , Amatavete S , Photisan N , Suriwong S , Noopetch P , Shanthachol T , et al. Differentiated service delivery for HIV treatment models in Thailand: a cross‐sectional assessment of real‐world implementation and uptake. Trop Med Int Health. 2023;28(5):374–383. 36938836 10.1111/tmi.13872

[62] Theresa H , Moses B , Otoyo T , Caroline F , Bhagawan S , Phayvieng P , et al. How home delivery of antiretroviral drugs ensured uninterrupted HIV treatment during COVID‐19: experiences from Indonesia, Laos, Nepal, and Nigeria. Glob Health: Sci Pract. 2021;9(4):978. 34933991 10.9745/GHSP-D-21-00168 PMC8691873

[63] Ibiloye O , Masquillier C , Jwanle P , Van Belle S , van Olmen J , Lynen L , et al. Community‐based ART service delivery for key populations in sub‐Saharan Africa: scoping review of outcomes along the continuum of HIV care. AIDS Behav. 2022;26(7):2314–2337. 35039936 10.1007/s10461-021-03568-3 PMC9162992

[64] Fujita M , Poudel KC , Green K , Wi T , Abeyewickreme I , Ghidinelli M , et al. HIV service delivery models towards ‘zero AIDS‐related deaths’: a collaborative case study of 6 Asia and Pacific countries. BMC Health Serv Res. 2015;15(1):176. 25902708 10.1186/s12913-015-0804-5 PMC4421992

[65] Benade M , Nichols BE , Fatti G , Kuchukhidze S , Takarinda K , Mabhena‐Ngorima N , et al. Economic evaluation of a cluster randomized, non‐inferiority trial of differentiated service delivery models of HIV treatment in Zimbabwe. PLOS Glob Public Health. 2023;3(3):e0000493. 36962960 10.1371/journal.pgph.0000493 PMC10021451

[66] Nichols BE , Cele R , Lekodeba N , Tukei B , Ngorima‐Mabhena N , Tiam A , et al. Economic evaluation of differentiated service delivery models for HIV treatment in Lesotho: costs to providers and patients. J Int AIDS Soc. 2021;24(4):e25692. 33838012 10.1002/jia2.25692 PMC8035675

[67] Guthrie T , Muheki C , Rosen S , Kanoowe S , Lagony S , Greener R , et al. Similar costs and outcomes for differentiated service delivery models for HIV treatment in Uganda. BMC Health Serv Res. 2022;22(1):1315. 36329450 10.1186/s12913-022-08629-4 PMC9635081

[68] Tran BX , Nguyen LH , Turner HC , Nghiem S , Vu GT , Nguyen CT , et al. Economic evaluation studies in the field of HIV/AIDS: bibliometric analysis on research development and scopes (GAPRESEARCH). BMC Health Serv Res. 2019;19(1):834. 31727059 10.1186/s12913-019-4613-0 PMC6854742

[69] HIV sensitive social protection: a review of Cambodia's social protection schemes for incorporating HIV sensitivity. Phnom Penh: UNDP, UNAIDS, National AIDS Authority, Ministry of Interior; 2013.

[70] Barennes H , Frichittavong A , Gripenberg M , Koffi P . Evidence of high out of pocket spending for HIV care leading to catastrophic expenditure for affected patients in Lao People's Democratic Republic. PLoS One. 2015;10(9):e0136664. 26327558 10.1371/journal.pone.0136664 PMC4556637

[71] Siregar AY , Tromp N , Komarudin D , Wisaksana R , van Crevel R , van der Ven A , et al. Costs of HIV/AIDS treatment in Indonesia by time of treatment and stage of disease. BMC Health Serv Res. 2015;15:440. 26424195 10.1186/s12913-015-1098-3 PMC4590258

[72] The socioeconomic impact of HIV at the household level in Cambodia. Phnom Penh: National AIDS Authority, United Nations; 2010.

