# Supplementary material for: Cost‐effectiveness analysis of a community‐based model for delivery of antiretroviral therapy to people with clinically stable HIV in Cambodia
Source: J Int AIDS Soc. 2025 Jul 7;28(Suppl 3):e26476. doi: 10.1002/jia2.26476 (PMC12234211; doi:10.1002/jia2.26476)
Supplement: Supplementary file 1 — Supporting information: CHEERS 2022 Checklist. Table S1: Univariate regression analysis of loss to follow‐up with outcomes and sociodemographic characteristics at baseline. Table S2: Computation of cost items at the health system and societal levels. [file JIA2-28-e26476-s001.pdf]

CHEERS 2022 Checklist

Topic	No.	Item	Location where item is reported
**Title**			
	1	Identify the study as an economic evaluation and specify the interventions being compared.	Page 1
**Abstract**			
	2	Provide a structured summary that highlights context, key methods, results, and alternative analyses.	Page 2
**Introduction**			
**Background and objectives**	3	Give the context for the study, the study question, and its practical relevance for decision making in policy or practice.	Page 3-4
**Methods**			
**Health economic analysis plan**	4	Indicate whether a health economic analysis plan was developed and where available.	Page 6
**Study population**	5	Describe characteristics of the study population (such as age range, demographics, socioeconomic, or clinical characteristics).	Page 4
**Setting and location**	6	Provide relevant contextual information that may influence findings.	Page 4
**Comparators**	7	Describe the interventions or strategies being compared and why chosen.	Page 4
**Perspective**	8	State the perspective(s) adopted by the study and why chosen.	Page 8
**Time horizon**	9	State the time horizon for the study and why appropriate.	Page 6
**Discount rate**	10	Report the discount rate(s) and reason chosen.	Not applicable
**Selection of outcomes**	11	Describe what outcomes were used as the measure(s) of benefit(s) and harm(s).	Page 5
**Measurement of outcomes**	12	Describe how outcomes used to capture benefit(s) and harm(s) were measured.	Page 5

Topic	No.	Item	Location where item is reported
**Valuation of outcomes**	13	Describe the population and methods used to measure and value outcomes.	Page 5
**Measurement and valuation of resources and costs**	14	Describe how costs were valued.	Page 7
**Currency, price date, and conversion**	15	Report the dates of the estimated resource quantities and unit costs, plus the currency and year of conversion.	Page 7
**Rationale and description of model**	16	If modelling is used, describe in detail and why used. Report if the model is publicly available and where it can be accessed.	Not applicable
**Analytics and assumptions**	17	Describe any methods for analysing or statistically transforming data, any extrapolation methods, and approaches for validating any model used.	Page 6-7
**Characterising heterogeneity**	18	Describe any methods used for estimating how the results of the study vary for subgroups.	Not applicable
**Characterising distributional effects**	19	Describe how impacts are distributed across different individuals or adjustments made to reflect priority populations.	Not applicable
**Characterising uncertainty**	20	Describe methods to characterise any sources of uncertainty in the analysis.	Page 6-8
**Approach to engagement with patients and others affected by the study**	21	Describe any approaches to engage patients or service recipients, the general public, communities, or stakeholders (such as clinicians or payers) in the design of the study.	Page 13
**Results**			
**Study parameters**	22	Report all analytic inputs (such as values, ranges, references) including uncertainty or distributional assumptions.	8-9
**Summary of main results**	23	Report the mean values for the main categories of costs and outcomes of interest and summarise them in the most appropriate overall measure.	9-10
**Effect of uncertainty**	24	Describe how uncertainty about analytic judgments, inputs, or projections affect findings. Report the effect of choice of discount rate and time horizon, if applicable.	9

Topic	No.	Item	Location where item is reported
**Effect of engagement with patients and others affected by the study**	25	Report on any difference patient/service recipient, general public, community, or stakeholder involvement made to the approach or findings of the study	Not applicable
**Discussion**			
**Study findings, limitations, generalisability, and current knowledge**	26	Report key findings, limitations, ethical or equity considerations not captured, and how these could affect patients, policy, or practice.	10-12
**Other relevant information**			
**Source of funding**	27	Describe how the study was funded and any role of the funder in the identification, design, conduct, and reporting of the analysis	13
**Conflicts of interest**	28	Report authors conflicts of interest according to journal or International Committee of Medical Journal Editors requirements.	12

From: Husereau D, Drummond M, Augustovski F, et al. Consolidated Health Economic Evaluation Reporting Standards 2022 (CHEERS 2022) Explanation and Elaboration: A Report of the ISPOR CHEERS II Good Practices Task Force. Value Health 2022;25. doi:10.1016/j.jval.2021.10.008

Table S1. Univariate regression analysis of loss-to-follow-up with outcomes and sociodemographic characteristics at baseline.

	Odds Ratio / Coefficient	SE	*p*-value
**Outcomes**			
ART adherence	1.14	0.13	0.21
Physical Health Component	0.85	0.06	0.03
Mental Health Component	1.05	0.09	0.56
**Sociodemographic**			
ART clinic setting	1.33	0.11	<0.001
Age groups	1.47	0.21	0.007
Gender groups	1.18	0.09	0.02
Marital status	1.13	0.08	0.10
Formal education	1.17	0.11	0.08

Employment	1.01	0.09	0.91
Family group size	1.01	0.24	0.95
Family with or without children	1.03	0.08	0.72
Duration living with HIV	1.78	0.17	<0.001
Duration receiving ART therapy	1.77	0.16	<0.001
Household monthly income (Riel)	96443.6	380385.7	0.80
Mode of Transportation to ART clinic	0.72	0.15	0.12
Travel time to ART clinic	0.78	0.06	0.001
Waiting time at ART clinic	0.09	0.05	0.05
Has comorbidity	1.32	0.12	0.003

Abbreviation: ART = antiretroviral therapy, SE = standard error

Table S2. Computation of cost items at the health system and societal levels.

Cost Item	Computation
**Health System Cost**	
Personnel salary – CAW[a]	Incentives, contributions to the National Social Security Fund, transport costs for distributing pre-packaged ARVs, and communication expenses provided to 82 CAWs each month throughout the project period.
Training cost[a,b]	For the training of CAWs in the CAD arm, and for the training of health workers at the ART clinics in the MMD arm, costs include venue booking, facilitator's fees, and per-diems for both trainers and trainees.
Operational cost for ART clinics in CAD[a]	Communication costs provided to the ART clinics in the CAD arm each month to support their communication with CAWs, and to organise bi-monthly meeting with CAWs during the project period.
**Societal Cost**	
OOPE incurred by people living with HIV and their caregivers[c]	Medical costs, including consultation, diagnostic, medications, and other medical expenses incurred by participants at the ART clinics, and non-medical costs, including travel, food, lodging and other expenses incurred by participants and their caregivers during their visits to the ART clinic. These costs were multiplied by the number of visits to the ART clinic over the project period to calculate the unweighted OOPE. Inverse probability weighting (IPW) was then applied to adjust for imbalances in baseline sociodemographic characteristics between the intervention and control arms, and the resulting weights were applied to the unweighted OOPE to generate weighted OOPE cost estimates.
OOPE incurred by CAWs[c]	Non-medical cost, including travel, food, lodging, and other expenses incurred by CAWs when they travelled to the ART clinics to collect ARVs and meet with the members in their CAGs, multiplied by the number of visits to the ART clinics

	and meetings with their members over the project period. Mean cost for all non-medical costs was multiplied by the number of CAWs to derive total OOPE for CAWs.
Productivity losses by people living with HIV and their caregivers[c]	Time spent by participants travelling to and at the ART clinic was multiplied by the number of visits to the ART clinic and their income over the project period to calculate the unweighted productivity cost. The IPW were then applied to the unweighted cost to derive the weighted productivity cost estimates.
Productivity losses by CAWs[c]	Time spent by CAWs travelling to and at the ART clinic, and time spent travelling to and at meetings with their members multiplied by the number of visits to the ART clinic and meetings with their members, respectively, over the project period. The total time was multiplied by their salary to derive productivity cost, and mean productivity cost was multiplied by the number of CAWs to derive total productivity losses for CAWs.

Abbreviations: ARV = anti-retroviral, ART = antiretroviral therapy, CAW = community action worker, CAD = community antiretroviral delivery, IPW = inverse probability weighting, OOPE = out-of-pocket expenditure
Source of cost data: [a]Khmer HIV/AIDS NGO Alliance (KHANA), [b]NCHADS, [c]Baseline and endline survey
